# Intrinsic Hormone-Like Molecules and External Root Resorption During Orthodontic Tooth Movement. A Systematic Review and Meta-Analysis in Preclinical *in-Vivo* Research

**DOI:** 10.3389/fphys.2018.00303

**Published:** 2018-03-28

**Authors:** Andreas Spoerri, Despina Koletsi, Theodore Eliades

**Affiliations:** ^1^Clinic of Orthodontics and Paediatric Dentistry, Center of Dental Medicine, University of Zurich, Zurich, Switzerland; ^2^London School of Hygiene and Tropical Medicine, University of London, London, United Kingdom

**Keywords:** root resorption, tooth movement, calcium, prostaglandin, thyroxin

## Abstract

**Background:** External root resorption constitutes an adverse effect of orthodontic treatment. The aim of the present meta-analysis was to identify the effect of induced intrinsic/ hormone-like molecules such as prostaglandins, interleukins and others on external root resorption after orthodontic tooth movement in experimental animals

**Methods:** An electronic database search of the literature was performed (Medline via PubMed, EMBASE, LILACS, and Open Gray). Search terms included root resorption, tooth movement and animal type. Risk of bias assessment was made using the SYRCLE guidelines for animal studies and reporting quality was assessed through ARRIVE. Random effects meta-analysis was performed for the outcome root resorption after orthodontic tooth movement.

**Results:** Of the 124 articles initially retrieved, 13 were eligible for inclusion in the systematic review, while only 2 were included in the quantitative synthesis. Five studies investigated the effect of Prostaglandin E2, four studies the effect of Thyroxine, two the effect of Calcium ions (Ca++), while the rest investigated Misoprostol, Interleukin-12 and Interleukin-4. Risk of Bias in all studies was judged to be high overall, while reporting quality was suboptimal. According to the quantitative synthesis, there was no difference in root resorption after orthodontic tooth movement when Prostaglandin E2 coupled with Ca++ was administered in comparison to no substance administration (SMD: 0.48 mm^2^; 95% CI: −0.22, 1.19; *p* = 0.18).

**Conclusions:** Overall, there was no evidence to suggest a variation in root resorption when Prostaglandin E2 and Ca++ were administered, while there is an overriding need for further high quality experimental studies to inform available evidence on the effect of intrinsic substances on external root resorption.

## Introduction

### Rationale

Apical and lateral external root resorption is considered an undesirable and unpredictable adverse effect of orthodontic treatment that may result in permanent loss of tooth structure. The etiology of root resorption (RR) is complex and largely unknown, but it most likely consists a multifactorial problem involving patient-related (individual biologic variability and genetic predisposition) and treatment-related (effect of mechanical forces) risk factors (Weltman et al., 2010).

Orthodontic treatment-related risk factors include magnitude and method of force application, direction of tooth movement, treatment duration, or amount of apical root displacement (Weltman et al., 2010; Topkara, 2011; Zahrowski and Jeske, 2011; Jatania et al., 2012; Topkara et al., 2012; Roscoe et al., 2015). However, clinical manifestation of RR in patients subjected to orthodontic treatment involving comparable mechanotherapy and duration was found to be highly variable (Poumpros et al., 1994). Moreover, RR has also been diagnosed in patients free of orthodontic mechanisms (Sogur et al., 2008). This highlights the fact that the presence of other factors is likely to be involved in the etiology of RR (Engstrom et al., 1988).

Individual susceptibility and genetic predisposition are major factors when considering the potential to present RR. A number of patient-related risk factors including tooth and root morphology, the severity of a malocclusion, patient age and sex constitute important predisposing parameters (Talic et al., 2006; Sogur et al., 2008).

One large category of substances that are involved in the etiology and development of RR are intrinsic factors which are known to regulate general metabolism, like hormones, trace elements and eicosanoids (Seifi et al., 2003, 2015; Bartzela et al., 2009). First, low level of calcium ions (Ca++) induces an increase in the secretion of parathyroid hormone (PTH) which mediates long-term changes in systemic Ca++ levels circulation by influencing osteoblasts and osteoclasts which are the primary orchestrators of bone turnover. Activation of osteoclast cells results in elevated levels of RANKL, a protein that plays a central role in the activity and formation of osteoclasts, which in turn has been involved in the development of RR (Seifi et al., 2015). Second, animal studies have shown that the administration of high doses of l-thyroxine increases bone resorption activity (Persson et al., 1989; Shirazi et al., 1999). Administration of low thyroxine doses has been shown to reduce the extent of RR both in humans and in animals (Poumpros et al., 1994). Third, is the relationship between RR are Prostaglandins (PGs). Animal studies have reported that the rate of orthodontic tooth movement is significantly increased after administration of prostaglandin injections (Boekenoogen et al., 1996). Bone remodeling is stimulated through increase in the numbers of osteoclasts (Sekhavat et al., 2002; Seifi et al., 2015).

### Objectives

Several investigations have been conducted with primary interest on external root resorption during orthodontic tooth movement in general and more specifically evaluating how local or systemic administration of hormone-like molecules might bear an effect on RR in animal populations. However, until now there is no known systematic approach to gather the available evidence with regard to the effect of these intrinsic factors on RR (Tyrovola and Spyropoulos, 2001; Bartzela et al., 2009; Diravidamani et al., 2012). Therefore, the aim of the present systematic review was to provide a synthesis of all published animal studies based on experimental data on the effect of induced factor/substance administration on external root resorption. A detailed database search and ensuing statistical analysis of individual study findings were performed (where indicated).

### Research question

The focused question of the present review was to assess whether administration of endogenous factors and hormone-like molecules such as PGs, Ca++, and thyroxin may present an effect on external root resorption after orthodontic tooth movement.

## Materials and methods

### Systematic review protocol

The Preferred Reporting Items for Systematic Reviews and Meta-Analyses were followed for reporting of this systematic review (Liberati et al., 2009; Moher et al., 2009).

#### Eligibility criteria

The following inclusion criteria were applied:

- Study design: Randomized or non-randomized experimental studies involving animals and including a comparison group were considered (one or more comparison groups).
- Population/Animal: Any type of animal undergoing orthodontic treatment forces.
- Interventions: Systemic/ local administration of intrinsic hormones/molecules during orthodontic tooth movement.
- Comparators: Other intrinsic hormones used as comparators, or placebo/control.
- Outcome measures: Difference in orthodontically induced root resorption.

Exclusion Criteria:

- *In vitro* studies.
- Animal studies without a comparison group.
- Animal studies involving administration of pharmaceutical/ exogenous hormones/molecules.

### Search strategy and date sources

Electronic search within the following databases was undertaken in October 5, 2017, while no language restrictions were applied: Medline via Pubmed, EMBASE and LILACS were searched. Moreover, gray literature was searched in Open Gray using the terms root resorption AND tooth movement. Hand searching of the reference lists of the retrieved full text articles was also conducted. Authors of original studies were contacted for data clarification where needed. Full search strategy employed in Medline via Pubmed is presented in Appendix 1 in Supplementary Material. Eligibility assessment, data extraction, reporting quality and Risk of Bias (RoB) assessment was implemented independently and in duplicate by two reviewers (AS and DK), while disagreements were resolved through discussion and after consultation with a third author (TE).

### Studies sections and data extraction

Data extraction was performed on standardized piloted forms by two independently working reviewers (AS and DK) who were not blinded to author identity and study origin. Titles and abstracts were examined first followed by full text screening of the potential for inclusion articles. Information was obtained from each included study on study design, population (type of animal), interventions, comparators and outcomes. In addition, information on type of tooth movement and duration was obtained.

#### Reporting quality

Reporting quality of the studies was assessed based on adherence to ARRIVE guidelines for reporting of animal studies (Animal Research: Reporting *in-vivo* Experiments). According to completeness of reporting, the reporting quality was judged as “clearly inadequate,” “possibly inadequate,” and “clearly adequate.” A grading system of 20 items contributed to the overall judgment of reporting quality (Kilkenny et al., 2012).

#### Risk of bias within studies

Risk of bias (RoB) in individual studies was assessed in line with the SYstematic Review Centre for Laboratory animal Experimentation (SYRCLE) RoB tool for animal studies (Hooijmans et al., 2014). In particular, the following 10 domains were considered: (1) Sequence generation, (2) Baseline characteristics, (3) Allocation concealment, (4) Random housing, (5) Blinding of researchers, (6) Random outcome assessment, (7) Blinding of outcome assessors, (8) Incomplete outcome data, (9) Selective outcome reporting, (10) Other sources of bias.

An overall assessment of the risk of bias was made for each included study (high, unclear, low). Studies with at least 1 item designated to be at high risk of bias were regarded as having an overall high risk of bias. Reports with unclear risk of bias for one or more key domains were considered to be at unclear risk of bias and studies with low risk of bias in all domains were rated as low risk of bias.

### Data analysis

#### Summary measures and data synthesis

Clinical homogeneity of included studies was assessed through the examination of individual trial settings, eligibility criteria, interventions, experimental conditions and observation time. Statistical heterogeneity was examined through visual inspection of the confidence intervals (CIs) for the estimated treatment effects on forest plots. Also, a chi-square test was applied to assess heterogeneity; a *p*-value below the level of 10% (*p* < 0.1) was considered indicative of significant heterogeneity (Higgins et al., 2003). I^2^ test for homogeneity was also undertaken to quantify the extent of heterogeneity.

Random effects meta-analyses were conducted as they were considered more appropriate to better approximate expected variations in individual experimental settings. Treatment effects were calculated through pooled standardized mean differences (SMD) in root resorption related parameters with associated 95% Confidence Intervals (95% CIs) and Prediction Intervals where applicable (at least 3 trials needed).

#### Risk of bias across studies

If more than 10 studies were included in meta-analysis, publication bias was to be explored through standard funnel plots.

#### Additional analyses

Sensitivity analyses were pre-determined to explore and isolate the effect of studies with high risk of bias on the overall treatment effect if both high and lower risk of bias studies were included.

## Results

### Flow diagram and search details

**Figure 1:**
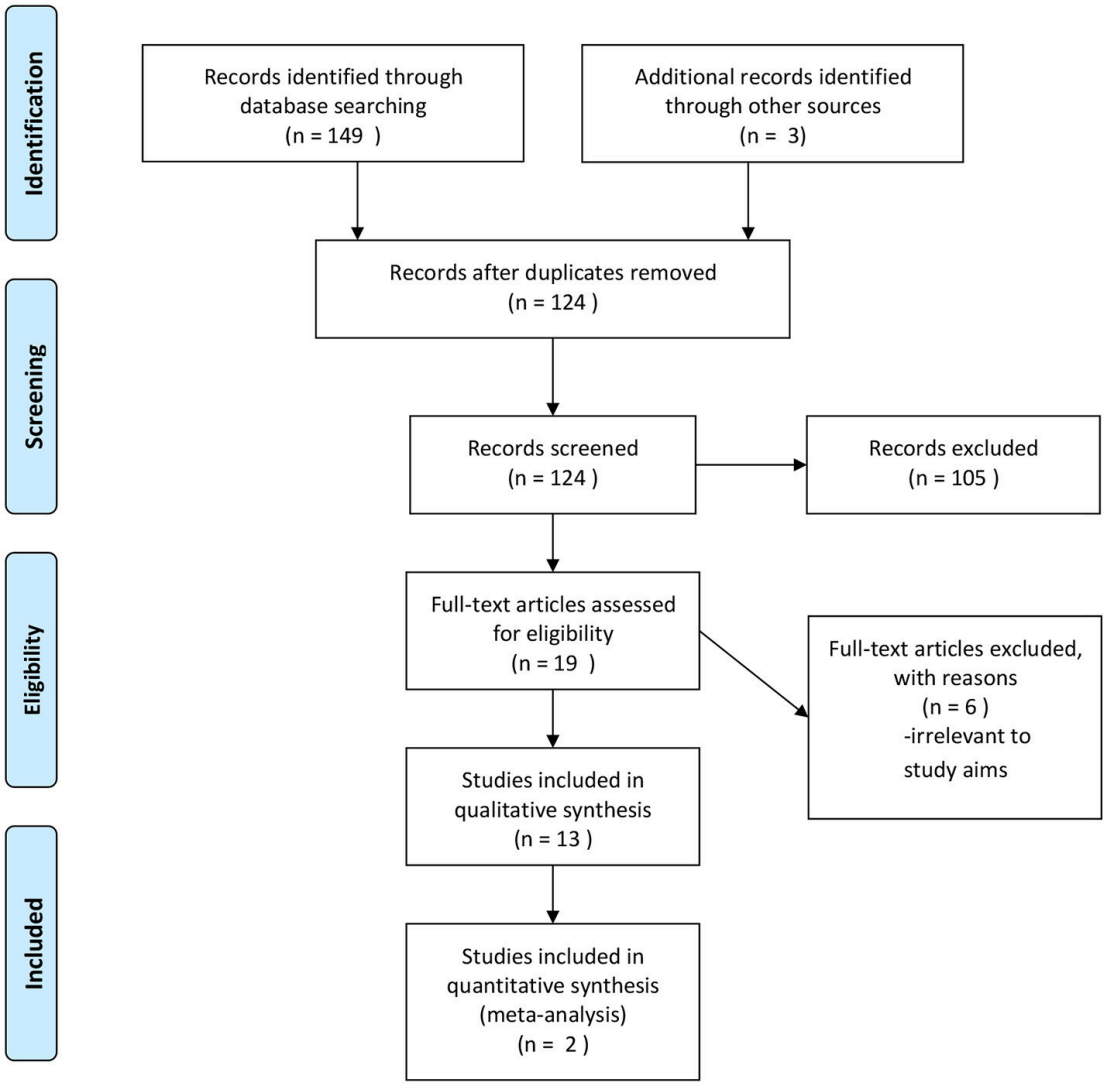
Flow diagram of study selection. From initial search to inclusion for synthesis.

The flow diagram of the study selection process is shown in Figure 1. The electronic search identified 149 articles, while records of 3 articles were identified after hand searching of the included for full-text evaluation studies. After the review of the abstracts and the full text manuscripts 13 studies were deemed eligible for inclusion in the review, while two where eligible for quantitative evaluation.

### Study selection and characteristics

Of the 13 studies, all were regarded as non-randomized. The most frequently used animals were rats (11 studies), whereas in two studies mice were used for the experiments (Yoshimatsu et al., 2012; Hakami et al., 2015). The sample sizes ranged from 24 (Seifi et al., 2003) to 132 (Boekenoogen et al., 1996) (min-max). Four studies each originated from the United States (Goldie and King, 1984; Poumpros et al., 1994; Leiker et al., 1995; Boekenoogen et al., 1996) and from Iran (Shirazi et al., 1999; Sekhavat et al., 2002; Seifi et al., 2003, 2015). Two studies originated from Japan (Yoshimatsu et al., 2012; Hakami et al., 2015) and one each from Turkey (Baysal et al., 2010), México (Vazquez-Landaverde et al., 2002), and Norway (Brudvik and Rygh, 1991).

#### Animal population

Four of the included studies used Wistar rats for their experiments (Brudvik and Rygh, 1991; Seifi et al., 2003, 2015; Baysal et al., 2010), whereas seven studies used Sprague-Dawley rats (Goldie and King, 1984; Poumpros et al., 1994; Leiker et al., 1995; Boekenoogen et al., 1996; Shirazi et al., 1999; Sekhavat et al., 2002; Vazquez-Landaverde et al., 2002). In two studies the experiments were done in mice (Yoshimatsu et al., 2012; Hakami et al., 2015). Most of the studies within this review used animals around 8 weeks old (Leiker et al., 1995; Boekenoogen et al., 1996; Vazquez-Landaverde et al., 2002; Seifi et al., 2003, 2015; Baysal et al., 2010; Yoshimatsu et al., 2012). In one study the animals were between 10 and 12 weeks old (Hakami et al., 2015). One study used 42 days old rats (Poumpros et al., 1994), whereas four studies didn't stated the age of the animals (Goldie and King, 1984; Brudvik and Rygh, 1991; Shirazi et al., 1999; Sekhavat et al., 2002; Table 1).

**Table 1:** Characteristics of included studies.

**Author, year**	**Species**	**Sex**	**Age**	**Weight**	**Sample**	**Interventions**	**Appliance (Material)**	**Location**		**Force**	**Experiment duration**
Seifi et al., 2015	WR	M	6–8 weeks	230–300 g	64	(G1) OA and TX, (G2) OA and PGE2, (G3) OA and CaGluconate, (G4) OA and PGE2 and Ca++, (G5) OA and TX and PGE2, (G6) OA and TX and Ca++, (G7) OA and PGE2 and Ca++ and TX, (G8) control (OA and distilled water)	Closed coil spring (NiTi)	Upper right first molar	Upper right incisor	60 g	21 days
Baysal et al., 2010	WR	M	7–8 weeks	132 (±12.6 g)	28	(G1) control with OA, (G2) control (G3) OA and TX, (G4) OA and DX, (G5) TX, (G6) DX	Closed coil spring (NiTi)	Upper right first molar	Incisors	50 g	14 days
Seifi et al., 2003	WR	M	8 weeks	230–300 g	24	(G1) control (saline injection and OA), (G2) normal (no injection, no OA), (G3) OA and PGE2, (G4) OA and Ca++ and PGE2	Closed coil spring (NiTi)	Upper right first molar	Upper right incisor	60 g	21 days
Sekhavat et al., 2002	SDR	M	NM	250 g (±20 g)	64	(G1) OA and 2.5 μg/kg MP, (G2) OA and 5.0 μg/kg MP, (G3) OA and 10.0 μg/kg MP, (G4) OA and 25.0 μg/kg MP, (G5) OA and 50.0 μg/kg MP, (G6) OA and 100.0 μg/kg MP, (G7) control (no OA), (G8) control with OA	Closed coil spring (NiTi)		Upper right incisor	60 g	14 days
Vazquez-Landaverde et al., 2002	SDR	M	8 weeks	250 g (±18 g)	80	(G1) control, (G2) C+TH oral, (G3) OA only, (G4) OA and TH intraperitoneal, (G5) OA and TH oral	Wire loop (NM)	Upper left first molar	Maxillary incisors	50 g	10 days
Shirazi et al., 1999	SDR	M	NM	240–280 g	50	(G1) no intervention, (G2) OA and saline, (G3) OA and 5 μg/kg bw TX, G4) OA and 10 μg/kg bw TX, (G5) OA and 20 μg/kg bw TX	Closed coil spring (NiTi)	Upper first molar	Maxillary left incisor	60 g	16 days
Boekenoogen et al., 1996	SDR	M	8 weeks	NM	132	(G1) control (no OA), (G2) OA 2 weeks, (G3) OA 4 weeks, (G4-11) 2 weeks period with different concentrations and injections timepoint, (G12-19) 4 weeks period with different concentrations and injections timepoint	Closed coil spring (NiTi)	Upper first molar	Maxillary incisor	60 g	G2, G4 to 11: 14 days; G 3, G12 to 19: 28 days
Leiker et al., 1995	SDR	M	8 weeks	225–250 g	127	(G1) control (no OA), (G2) OA 2 weeks, (G3) OA 4 weeks, (G4-11) 2 weeks period with different concentrations and injections timepoint, (G12-19) 4 weeks period with different concentrations and injections timepoint	Closed coil spring (NiTi)	Upper first molar	Maxillary incisor	60 g	G2, G4 to 11: 14 days; G 3, G12 to 19: 28 days
Brudvik and Rygh, 1991	WR	M	NM	165 g (±20 g)	25	(G1) no OA and PGE2 injection, (G2) OA and PGE2 injection (3 days), (G3) OA and PGE2 injection (7 days), (G4) OA and PGE2 injection (10 days), (G5) OA 3 (days), G6) OA (7 days), G7) OA (10 days), G8) no OA, no injection	Closed coil spring (NiTi)	Upper first molar	Incisor	50 g	(G1, G2, and G5): 3 days, G3 and G6: 7 days, G4 and G7: 10 days
Goldie and King, 1984	SDR	F	NM	NM	35	(G1) non-lactating animals on a control diet a: 7 days, b: 4 days, c: 7 days, d: 10 days, e: 14 days, (G2) lactating animals on a calcium-deficient diet and OA a: 7 days, b: 4 days, c: 7 days, d: 10 days, e: 14 days	Closed coil spring (NiTi)	Upper first molar	Maxillary incisor	60 g	G1a and c, G2a and c: 7 days; G1b, G2b: 4 days; G1d, G2d: 10 days; G1e, G2e: 10 days
Poumpros et al., 1994	SDR	M	6 weeks	140 g	48	(G1) normal, (G2) control with OA, (G3) OA and TX	Active spring (Australian)		Maxillary incisors	50 g	10 days
Yoshimatsu et al., 2012	C57BL6/J mice	M	8 weeks	NM	32	(G1) control (no OA, no injection), (G2) PBS every other day, (G3) 0.015 μg/day of IL-12, (G4) 0.15 μg/day of IL-12, (G5) 1.5 μg/day of IL-12	Closed coil spring (NiTi)	Upper left first molar	Maxillary incisors	10 g	12 days
Hakami et al., 2015	C57BL6/J mice	M	10–12 weeks	NM	NM	(G1) control (no OA, no injection), (G2) PBS every other day, (G3) 0.015 μg/day of IL-4, (G4) 0.15 μg/day of IL-4, (G5) 1.5 μg/day of IL-4	Closed coil spring (NiTi)	Upper left first molar	Upper anterior alveolar bone	10 g	12 days

*G, group, WR, Wistar rats; SDR, Sprague-Dawley rats; F, female; M, male; NM, not mentioned, PGE2, Prostaglandine; Ca++, calcium ions; TX, thyroxine; DX, doxycycline, MP, misoprostol; OA, orthodontic appliance, IL, interleukin*.

#### Interventions

Five studies investigated the effect on RR of Prostaglandin E2 (PGE 2) (Brudvik and Rygh, 1991; Leiker et al., 1995; Boekenoogen et al., 1996; Seifi et al., 2003, 2015), four studies the effect of Thyroxine (Poumpros et al., 1994; Shirazi et al., 1999; Vazquez-Landaverde et al., 2002; Baysal et al., 2010), two studies the effect of Ca++ (Goldie and King, 1984; Seifi et al., 2015), and one on misoprostol (Sekhavat et al., 2002), one on Interleukin-12 (IL-12) (Yoshimatsu et al., 2012) and one on Interleukin-4 (IL-4) (Hakami et al., 2015).

The orthodontic appliance used for tooth movement in most studies was a closed coil spring of NiTi material. Only one study used a wire loop (Vazquez-Landaverde et al., 2002). The springs were attached in the upper jaw either between first molars and an incisor or between the two incisors. The method of attachment was a ligature wire or a composite. The initial forces were mostly measured with a gauge ranged between 10 and 60 g (Table 1).

#### Outcomes

Root resoption was measured with varying methods/modalities across the investigated studies. In six studies the amount of root resorption was indicated in percentage (Goldie and King, 1984; Poumpros et al., 1994; Vazquez-Landaverde et al., 2002; Baysal et al., 2010; Yoshimatsu et al., 2012; Hakami et al., 2015) whereas in four studies RR was measured in distance (Sekhavat et al., 2002) or in area (Seifi et al., 2003, 2015). In two studies no numbers of the amount of RR were indicated (Leiker et al., 1995; Shirazi et al., 1999). In most studies the first maxillary molar was used as the unit of analysis. The mesial root of the upper first molar was investigated in eight studies (Goldie and King, 1984; Leiker et al., 1995; Boekenoogen et al., 1996; Shirazi et al., 1999; Sekhavat et al., 2002; Seifi et al., 2003, 2015; Baysal et al., 2010) while in two the distobuccal root was evaluated (Yoshimatsu et al., 2012; Hakami et al., 2015). One study assessed the middle or the distobuccal root (Brudvik and Rygh, 1991).

In addition, tooth movement induced by orthodontic appliances was measured in the majority of the studies as an exploratory outcome (Goldie and King, 1984; Poumpros et al., 1994; Leiker et al., 1995; Shirazi et al., 1999; Sekhavat et al., 2002; Baysal et al., 2010; Yoshimatsu et al., 2012; Hakami et al., 2015; Seifi et al., 2015).

#### Reporting quality of included studies

**Figure 2:**
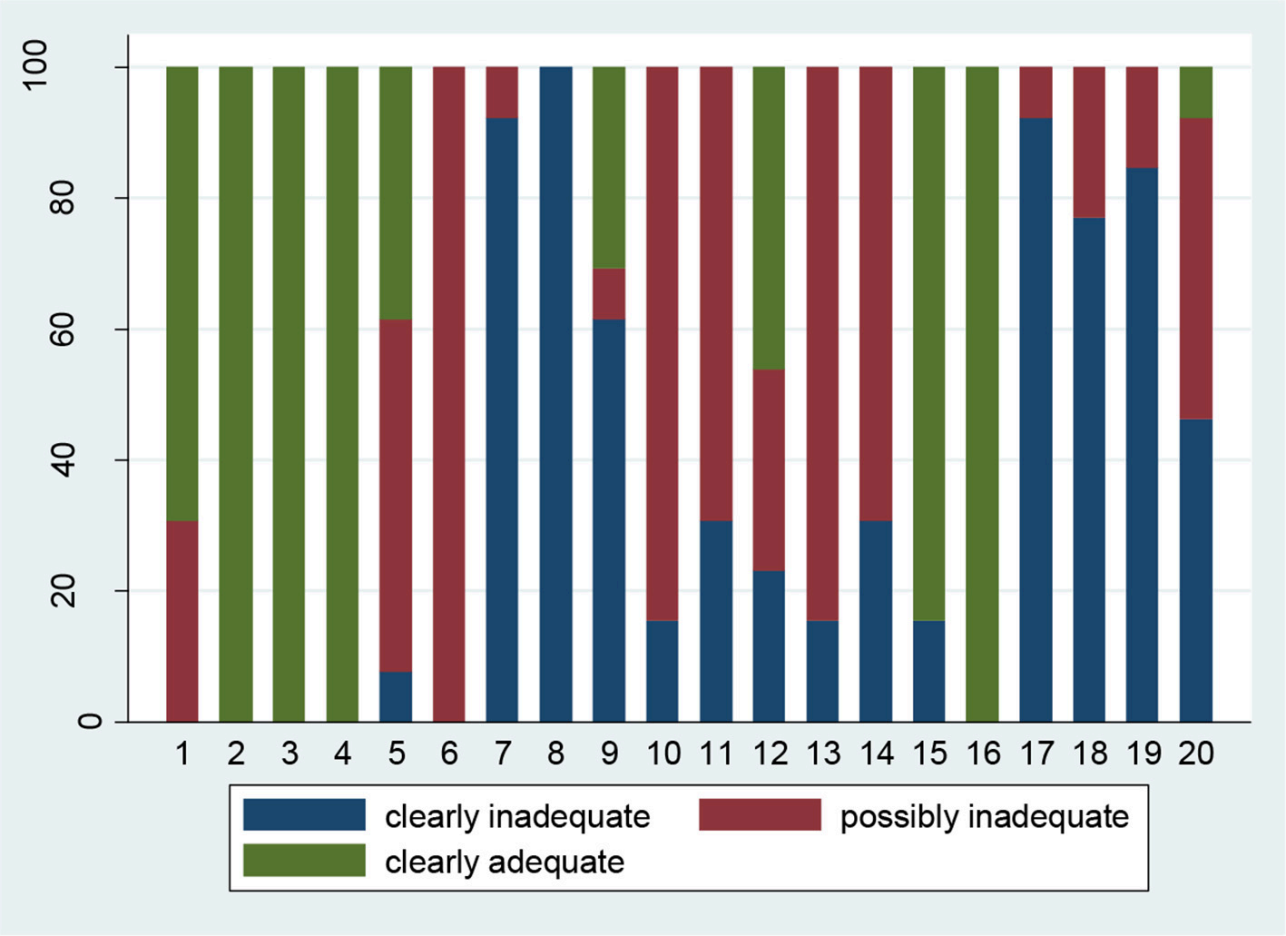
Summary (in percentage) of reporting across ARRIVE items. Specific ARRIVE domains corresponding to items can be found in Appendix Table 1 in Supplementary material.

Reporting quality criteria of the studies were assessed according to the ARRIVE criteria (Appendix Table 1 in Supplementary Material; Figure 2). Twenty items were evaluated. The identified studies did not clearly report information regarding experimental procedures, experimental animals, the sample size and animal allocation. Domains with optimal reporting pertained abstract, introduction, objectives and outcomes together with precision of estimation.

### Synthesized findings

#### Effects of interventions, meta-analyses, and additional analyses

In all studies an orthodontic appliance was put in place to simulate orthodontic tooth movement. Root resorption was measured on the maxillary first molar in all but two studies (Poumpros et al., 1994; Vazquez-Landaverde et al., 2002). When individual studies were investigated, Prostaglandin E2 was found to present an increase in root resorption after orthodontic tooth movement (Brudvik and Rygh, 1991; Leiker et al., 1995; Boekenoogen et al., 1996; Seifi et al., 2003, 2015). However, this was not the case for thyroxine (Poumpros et al., 1994; Shirazi et al., 1999; Vazquez-Landaverde et al., 2002; Baysal et al., 2010; Seifi et al., 2015), calcium deficient diet during lactation (Goldie and King, 1984) and interleukins (IL-4, IL-12) (Yoshimatsu et al., 2012; Hakami et al., 2015).

**Figure 3:**
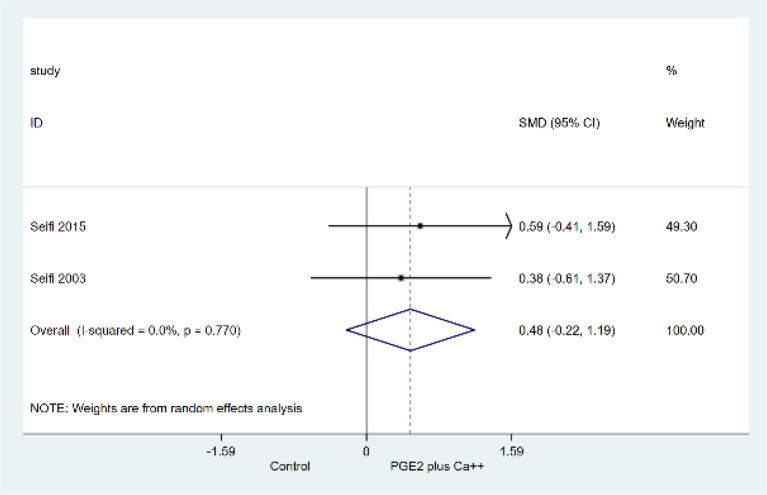
Random effects meta-analysis for the effect of Prostaglandin E2 coupled with Calcium ions (Ca++) vs. control (no substance administration) on external root resorption after orthodontic tooth movement.

Figure 3 presents the forest plot with the results from random effects meta-analysis with regard to the effect of PGE2 plus Ca on root resorption. There was no evidence to support α variation in root resorption after orthodontic tooth movement when Prostaglandin E2 coupled with Ca++ was administered in comparison to no substance administration (SMD: 0.48 mm^2^; 95%CI: −0.22, 1.19; *p* = 0.18). There was no evidence of statistical heterogeneity (I2 = 0.0%; chi-squared: *p* = 0.77).

#### Risk of bias across studies

Exploring for publication bias either statistically or graphically was not undertaken as no more than 2 studies were included in an individual meta-analysis.

### Risk of bias of included studies

**Figure 4:**
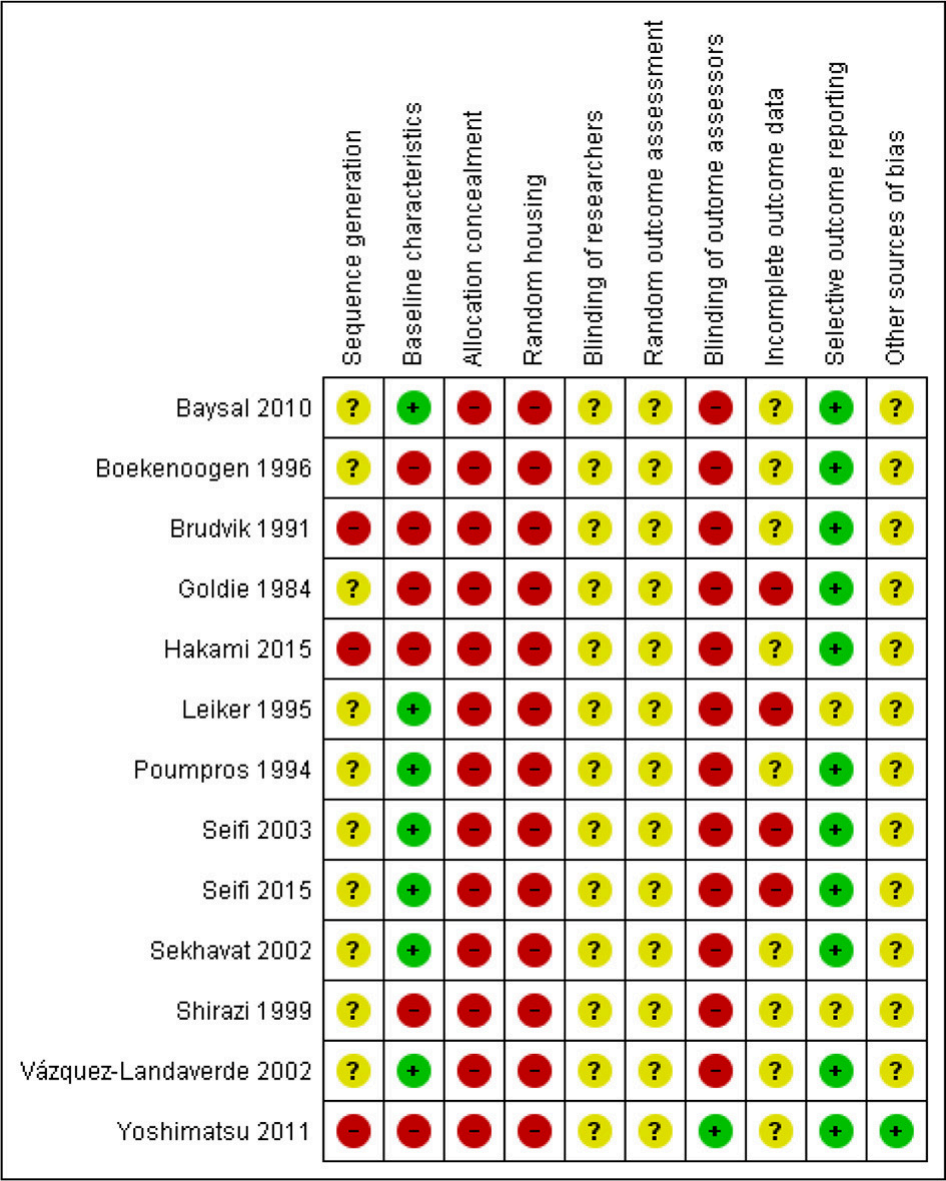
Risk of bias summary: review authors' judgments about each risk of bias item for each included study. Green circle denotes low risk of bias, yellow is unclear and red circle is high risk of bias.

Risk of bias (RoB) within the studies was assessed according to the SYRCLE guidelines. An overall assessment of the risk of bias was made for each included study (high, unclear, low). RoB in all studies was judged to be high risk of bias (Figure 4). Details on the reporting of randomization and allocation concealment strategies were insufficient in all of the included studies. A similar trend was detected also for items pertaining to blinding/masking of the personnel involved or the outcome assessor. Only one study reported blinding of the assessor (Yoshimatsu et al., 2012). Eleven studies had a low risk of reporting bias as they clearly provided sufficient details to allow for the assessment of study outcomes and potential discrepancies.

## Discussion

### Summary of main findings

In the present review, the effect of intrinsic hormone-like molecules on external root resorption during experimental orthodontic tooth movement was examined. To the best of our knowledge, this review is the first to systematically appraise the existing data about intrinsic substances affecting root resorption.

One of the most commonly investigated factors for its effect on RR are PGs. PGs are sysnthesized by cyclooxygenase from archdonic acid. Besides their important role in inflammation, PGs are important for the orthodontic treatment. They stimulate bone resorption by increasing the amount of osteoclasts, while they have been also reported to stimulate RR with varing prevalence (Brudvik and Rygh, 1991; Leiker et al., 1995; Boekenoogen et al., 1996; Sekhavat et al., 2002). Brudvik et al. (Brudvik and Rygh, 1991) was the first author who investigated RR in connection with local injection of PGE_2_ and found a trend toward increasing amounts of root resorption on the teeth where PGE_2_ injections were performed. These results were confirmed by other studies (Leiker et al., 1995; Boekenoogen et al., 1996; Seifi et al., 2003). Ca++ is yet another important regulator that is implicated in various physiologic mechanisms or processes. Ca++ homeostasis is regulated by different hormones such as thyroid hormones (thyroxine, calcitonin), parathyroid hormone (PTH), sex hormones (estrogens), and vitamines (e.g., vitamin D3) (Bartzela et al., 2009). Calcium-deficient diet during lactation has been shown to decrease bone density through induction of secondary hyperparathyroidism and has also been associated with reduced root resorption (Goldie and King, 1984). However, in this meta-analysis, when pooling the results of applicable studies regarding the combined use of PGE2 and Ca++, no significant effect on the amount of root resorption after application of orthodontic forces was recorded as compared to controls. Nevertheless, only two studies (Seifi et al., 2003, 2015) contributed to the overall estimate which presented high risk of bias and limited sample sizes.

Further, the thyroid hormone (i.e., thyroxine) plays a central role in normal growth and the development of vertebrate bones and has also been shown to reduce the extent of RR (Seifi et al., 2015). Poumpros et al. was the first to report that administration of low doses of thyroxin in animals appears to reduce force-induced RR (Poumpros et al., 1994). These date were confirmed by the findings of Shirazi et al. (1999) and Seifi et al. (2015), while Vazquez-Landaverde et al. (2002) have revealed the protective role of low doses of thyroxin on the root surface during orthodontic tooth movement in patients that present spontaneous RR lesions. Baysal et al. (2010) suggested that systemic administration of lower doses of thyroxine may have an inhibitory effect on orthodontically induced resorptive activity.

The clinical importance of this study lies on the potential to identify endogenous molecules that may bear a significant role in regulating orthodontic tooth movement and external root resorption. This might prove beneficial for clinicians who would use this knowledge to achieve optimization in treatment procedures and elimination of adverse effects of orthodontic treatment. Concurrently, individualized treatment plannings would be favored and grounded on biologic backgrounds.

The results of the study are restricted to animal models and extrapolation to human clinical conditions is one of the major concerns of animal studies. All the experiments described were performed in rodents with the vast majority in rats. The physiologic and morphologic differences between human and rat periodontal ligament and alveolar bone should be taken into account when interpreting the results from animal research. Intrestingly, alveolar bone in rats shows no osteons and is denser than human alveolar bone. On the contrary, osteoid tissue along the alveolar bone surfaces is more abundant in human (Ren et al., 2004; Dutra et al., 2017). Further, it appears that the balance of Ca++ in rats is primarily regulated by intestinal absorption than by bone tissue, while structural dissimilarities in the arrangement of the periodontal fibers and the supporting structures have been recorded (Ren et al., 2004). However, small animals such as rats or mice are widely used in orthodontic research and beyond as they present significant advantages: short gestation period, low housing costs and easy handling which faciliates the use of conviniently large samples.

The ARRIVE guidelines (Kilkenny et al., 2012) have been endorsed since 2010 to ameliorate the standards of reporting in preclinical animal research and have been used by the present review to disclose the quality of reporting with regard to root resorption and endogenous elements. Although the guidelines have been used in other fields of dentistry (Berglundh and Stavropoulos, 2012), the need for improved and standardized approaches in reporting of RR after orthodontic tooth movement seems imperative. In addition, the SYRCLE tool (Hooijmans et al., 2014) was followed to assess the internal validity and risk of bias within individual studies. Overall, risk of bias was rated as high with selection and detection bias being the strongest contributing domains as no details about randomization or blinding of outcome assessors were reported. This may reveal significant shortcomings in the design and reporting of animal studies which in turn may bear an effect on laboratory research waste. The SYRCLE risk of bias tool used in this systematic review was a first step toward transparency in the reporting of resorption related factors during orthodontic tooth movement that will presumably assist in improving design, conduct and analysis of future work.

### Limitations

This review is not free of limitations. The effect of low or unclear risk of bias studies could not be isolated due to the scarcity of the available studies and as such the interpretation of the included studies should be considered with caution. Furthermore, publication bias was not examined as only two studies were available for quantitative synthesis.

## Conclusion

There is no solid evidence to determine the effect of intrinsic hormone-like molecules on external root resorption after orthodontic tooth movement. There is an increasing need for further standardized and high quality experimental studies to fill knowledge gaps and inform future clinical research.

## Author contributions

DK and TE had the idea of the systematic review. DK did the experimental design. AS and DK did the literature search. Disagreements were resolved through discussion and after consultation with TE. DK did the statistical analysis. AS and DK wrote the manuscript. AS, DK and TE edited the manuscript. All authors read and approved the final manuscript before submission.

### Conflict of interest statement

The authors declare that the research was conducted in the absence of any commercial or financial relationships that could be construed as a potential conflict of interest.
