# Supplementary material for: Intrinsic Hormone-Like Molecules and External Root Resorption During Orthodontic Tooth Movement. A Systematic Review and Meta-Analysis in Preclinical in-Vivo Research
Source: Front Physiol. 2018 Mar 28;9:303. doi: 10.3389/fphys.2018.00303 (PMC5882845; doi:10.3389/fphys.2018.00303)
Supplement: Supplementary file 1 [file Table1.docx]

**Appendix 1.**

MEDLINE search

Limits: no language restriction applied

Publication date: no restriction

Search Builder: ‘All Fields’

Four consecutive searches combined with "AND" Boolean operator, using “OR” between free text terms or keywords:

1. mice

2. mouse

3. rat

4. rat*

5. rodent

6. pig

7. animal

8. dog

9. cat

10. rabbit

11. monkey

12. 1 OR 2 OR 3 OR 4 OR 5 OR 6 OR 7 OR 8 OR 9 OR 10 OR 11

13. root resorption

14. root resorpt*

15. resorption

16. tooth movement

17. teeth movement

18. tooth move*

19. teeth move*

20. 13 OR 14 OR 15 OR 16 OR 17 OR 18 OR 19

21. thyroid hormone

22. parathyroid hormone

23. prostaglandin

24. calcium

25. calcium gluconate

26. 21 OR 22 OR 23 OR 24 OR 25

27. 12 AND 20 AND 26

Appendix Table 1.

|  | **ARRIVE statements** | **Seifi 2015** | **Baysal 2010** | **Seifi 2003** | **Sekhavat 2002** | **Vázquez-Landaverde 2002** | **Shirazi 1999** | **Boekenoogen 1996** | **Leiker 1995** | **Brudvik 1991** | **Goldie 1984** | **Poumpros 1994** | **Yoshimatsu 2011** | **Hakami 2015** |
| --- | --- | --- | --- | --- | --- | --- | --- | --- | --- | --- | --- | --- | --- | --- |
| 1 | Title | 2 | 2 | 2 | 1 | 2 | 1 | 2 | 1 | 2 | 2 | 1 | 2 | 2 |
| 2 | Abstract | 2 | 2 | 2 | 2 | 2 | 2 | 2 | 2 | 2 | 2 | 2 | 2 | 2 |
| 3 | Introduction | 2 | 2 | 2 | 2 | 2 | 2 | 2 | 2 | 2 | 2 | 2 | 2 | 2 |
| 4 | Objectives | 2 | 2 | 2 | 2 | 2 | 2 | 2 | 2 | 2 | 2 | 2 | 2 | 2 |
| 5 | Ethical statement | 2 | 2 | 2 | 0 | 1 | 0 | 0 | 2 | 0 | 0 | 0 | 2 | 0 |
| 6 | Study design | 1 | 1 | 1 | 1 | 1 | 1 | 1 | 1 | 1 | 1 | 1 | 1 | 1 |
| 7 | Experimental procedures | 1 | 0 | 0 | 0 | 0 | 0 | 0 | 0 | 0 | 0 | 0 | 0 | 0 |
| 8 | Experimental animals | 0 | 0 | 0 | 0 | 0 | 0 | 0 | 0 | 0 | 0 | 0 | 0 | 0 |
| 9 | Housing and husbandry | 1 | 2 | 0 | 0 | 2 | 0 | 0 | 0 | 0 | 0 | 0 | 2 | 2 |
| 10 | Sample size | 1 | 1 | 1 | 1 | 1 | 1 | 1 | 1 | 1 | 1 | 1 | 0 | 0 |
| 11 | Allocationg animals to experimental groups | 1 | 1 | 1 | 1 | 1 | 1 | 0 | 1 | 0 | 1 | 1 | 0 | 0 |
| 12 | Experimental outcomes | 1 | 2 | 1 | 1 | 2 | 2 | 2 | 2 | 2 | 1 | 0 | 0 | 0 |
| 13 | Statistical methods | 1 | 1 | 1 | 1 | 1 | 1 | 1 | 1 | 1 | 0 | 1 | 1 | 0 |
| 14 | Baseline data | 1 | 1 | 1 | 1 | 1 | 1 | 0 | 1 | 1 | 0 | 1 | 0 | 0 |
| 15 | Numbers analysed | 2 | 2 | 2 | 2 | 2 | 2 | 2 | 2 | 2 | 2 | 2 | 0 | 0 |
| 16 | Outcomes and estimation | 2 | 2 | 2 | 2 | 2 | 2 | 2 | 2 | 2 | 2 | 2 | 2 | 2 |
| 17 | Adverse events | 0 | 0 | 0 | 0 | 0 | 0 | 1 | 0 | 0 | 0 | 0 | 0 | 0 |
| 18 | Interpretation/scientific implications | 0 | 1 | 0 | 0 | 0 | 1 | 0 | 0 | 1 | 0 | 0 | 0 | 0 |
| 19 | Generalisability/translation | 0 | 1 | 0 | 0 | 0 | 0 | 0 | 0 | 1 | 0 | 0 | 0 | 0 |
| 20 | Funding | 0 | 1 | 0 | 0 | 1 | 0 | 1 | 0 | 1 | 1 | 0 | 2 | 1 |

0=clearly inadequate, 1=possibly inadequate, 2=clearly adequate
